# Extracellular Protein Aggregates Colocalization and Neuronal Dystrophy in Comorbid Alzheimer’s and Creutzfeldt–Jakob Disease: A Micromorphological Pilot Study on 20 Brains

**DOI:** 10.3390/ijms22042099

**Published:** 2021-02-20

**Authors:** Nikol Jankovska, Tomas Olejar, Radoslav Matej

**Affiliations:** 1 Department of Pathology and Molecular Medicine, Third Faculty of Medicine, Charles University and Thomayer University Hospital, Videnska 800, 4-Krc, 14059 Prague, Czech Republic; tomas.olejar@ftn.cz (T.O.); radoslav.matej@ftn.cz (R.M.); 2 Department of Pathology, First Faculty of Medicine, Charles University, and General University Hospital, 4-Krc, 14059 Prague, Czech Republic; 3 Department of Pathology, Third Faculty of Medicine, Charles University, and University Hospital Kralovske Vinohrady, 4-Krc, 14059 Prague, Czech Republic

**Keywords:** Creutzfeldt–Jakob disease, Alzheimer’s disease, Aβ, prion protein, tau protein, colocalization, plaques, confocal microscopy

## Abstract

Alzheimer’s disease (AD) and sporadic Creutzfeldt–Jakob disease (sCJD) are both characterized by extracellular pathologically conformed aggregates of amyloid proteins—amyloid β-protein (Aβ) and prion protein (PrP^Sc^), respectively. To investigate the potential morphological colocalization of Aβ and PrP^Sc^ aggregates, we examined the hippocampal regions (archicortex and neocortex) of 20 subjects with confirmed comorbid AD and sCJD using neurohistopathological analyses, immunohistochemical methods, and confocal fluorescent microscopy. Our data showed that extracellular Aβ and PrP^Sc^ aggregates tended to be, in most cases, located separately, and “compound” plaques were relatively rare. We observed PrP^Sc^ plaque-like structures in the periphery of the non-compact parts of Aβ plaques, as well as in tau protein-positive dystrophic structures. The AD ABC score according to the NIA-Alzheimer’s association guidelines, and prion protein subtype with codon 129 methionine–valine (M/V) polymorphisms in sCJD, while representing key characteristics of these diseases, did not correlate with the morphology of the Aβ/PrP^Sc^ co-aggregates. However, our data showed that PrP^Sc^ aggregation could dominate during co-aggregation with non-compact Aβ in the periphery of Aβ plaques.

## 1. Introduction

Deposits of extracellular protein aggregates are diagnostic findings for two separate neurodegenerative diseases, i.e., Alzheimer’s (AD) and Creutzfeldt–Jakob diseases (CJD) [1,2]. Amyloid-β peptide (Aβ) is a main defining component of Aβ plaques (also called amyloid or senile plaques) observed in AD [3,4]. These extracellular deposits arise from the amyloidogenic cleavage of an integral membrane protein, called amyloid precursor protein (APP), by beta-site APP cleaving enzyme 1 (β-secretase/BACE 1), which is found on neuronal membranes [5]. In addition to APP and BACE 1, the physiological isoform of the prion protein (PrP^C^) is also found on the outer surface of neuronal membranes; it is attached to the membrane via a glycosylphosphatidylinositol (GPI) anchor [6].

A full understanding of the physiological role of Aβ and PrP^C^ remains elusive. Briefly, Aβ plays a critical role in brain development, neuronal migration, and synaptic plasticity [7]. Additionally, Aβ interacts with Cu and Zn ions, e.g., rising copper levels increase the amount of APP on cell surfaces [8]; therefore, the increased presence of Cu ions mediates the precipitation of Aβ deposits [9]. Data from murine gene knock-outs suggest a functional role for PrP^C^ in myelination maintenance in adults, neuronal plasticity in adults, and the circadian rhythm [10].

Currently, molecular interactions between Aβ and PrP, in either physiological or pathological forms, are being widely investigated, with interactions between oligomeric Aβ and physiological PrP^C^ receiving particular attention [11]. Other studies have focused on transfected SH-SY5Y neuroblastoma cells, cellular overexpression of PrP, decreased amyloidogenic cleavage of APP, and silencing of PrP^C^ genes in N2A cells, via the increased secretion of Aβ [12].

It has also been shown that the scrapie isoform of prion protein (PrP^Sc^) could alter APP processing through stimulation of 3-phosphoinositide-dependent protein kinase 1 (PDK1 or PDPK1) and the inhibition of alpha-secretase activity, which could lead to enhanced β-secretase processing accompanied by increased Aβ production [13]. There is another connection between these two proteins; as γ-secretase cleaves the residual APP C-terminal fragment, thus creating Aβ, it leaves behind the amyloid intracellular domain (AICD) [14], which according to recent research, controls the expression of PrP^C^ [15].

Membrane PrP^C^ acts as a receptor for Aβ oligomers; this feature helps explain its involvement in AD development [16]. Nonetheless, both AD and CJD have been described as having very similar dystrophic neurites containing mostly autophagic vacuoles and autophagosomes [17].

Even though microtubule-associated protein (MAP) tau mainly forms intracellular amyloid aggregates in AD, its functional interaction with PrP^C^ and PrP^Sc^ has also been reported. PrP^C^ probably plays a critical role related to Aβ and tau protein in AD development [18], with PrP^C^ acting as a mediator of synaptic dysfunction induced by tau protein [19]. It is not unreasonable to expect dystrophic neurites with hyperphosphorylated tau protein in neuritic amyloid plaques. As such, dystrophic neurites in plaque-like PrP^Sc^ structures that colocalize with Aβ would also not be unexpected. There is increasing evidence that more than one neurodegeneration in the brain is possible at the same time [20]. However, the precise interactions among crucial amyloidogenic proteins in the pathophysiology of neurodegenerations remain unclear. Moreover, there is only limited information related to the morphological interactions among these brain peptides during comorbid neurodegenerations.

In our pilot study, we evaluated using immunohistochemistry and confocal microscopy, the micromorphology of PrP^Sc^ colocalized with Aβ in dystrophic neurites with compound plaques in the brains of patients with comorbid Alzheimer’s and Creutzfeldt–Jakob disease [21].

## 2. Results

### 2.1. Confocal Microscopy Visualization

**Figure 1:**
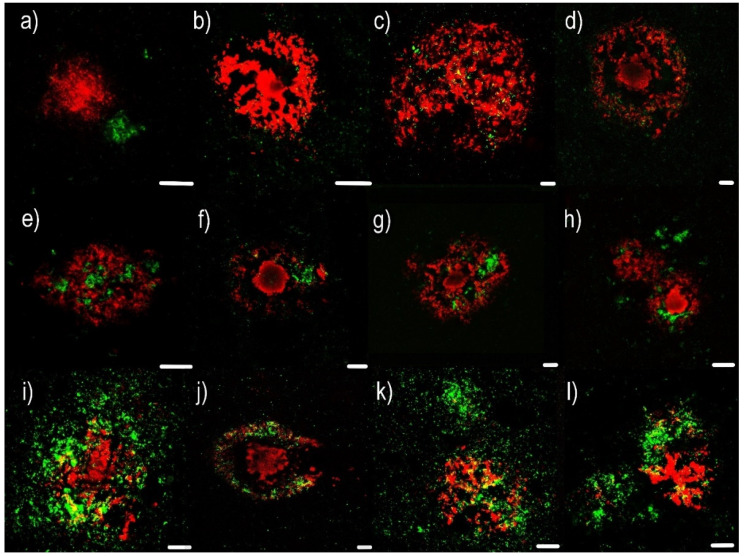
Immunofluorescence visualization of different types of anti-amyloid β-protein (Aβ) and prion protein (PrP^Sc^) compound plaques in comorbid Alzheimer’s (AD) and Creutzfeldt–Jakob diseases (CJD) cases. Primary antibodies: anti-PrP (rabbit recombinant monoclonal antibody) + anti-amyloid β-protein (mouse monoclonal antibody). The secondary antibody was conjugated with either Alexa Fluor^®^ 488 (anti-rabbit IgG; green) or Alexa Fluor^®^ 568 (anti-mouse IgG; red). Scale bars indicate 10 micrometers. Images come from the hippocampal region (archicortical parts). (**a**,**b**) Non-compound plaques: consisting of (**a**) Aβ diffuse or (**b**) cored neuritic plaques, and lacking co-aggregating PrP structures. Images come from a 75-year-old female suffering from sCJD + AD (A1B1C1), cerebral amyloid angiopathy (CAA) 0. (**c**,**d**) Minimal compound plaques: diffuse (**c**) or cored neuritic (**d**) Aβ plaques with punctate PrP aggregates. Images come from a 69-year-old female suffering from sCJD + AD (A2B2C1), CAA 0. (**e**–**h**) Central core deposits: Neuritic non-cored (**e**) or cored (**f**–**h**) Aβ plaques with distinct PrP^Sc^ aggregates in the center of the plaque. Images come from two patients—a 79-year-old man suffering from sCJD + AD (A2B2C2), CAA 0, + ARTAG + Fahr disease, and a 71-year-old female sCJD + AD (A2B1C2), CAA. (**i**,**j**) Diffuse compound plaques: these are (**i**) neuritic non-cored or (**j**) cored Aβ plaques where co-aggregation of PrP^Sc^ at the periphery in the area of the non-compact Aβ structures is evident. The samples come from a 64-year-old male suffering from sCJD + early-onset AD (A2B2C2), CAA 0 + Wernicke’s encephalopathy. (**k**,**l**) Diffuse “Yin-Yang” compound plaques: neuritic non-cored Aβ plaques having a prominent admixture of PrP^Sc^ co-aggregation predominantly localized at one pole of the plaque. The images come from a 70-year-old female patient sCJD + AD (A2B2C3) + CAA 0.

**Figure 2:**
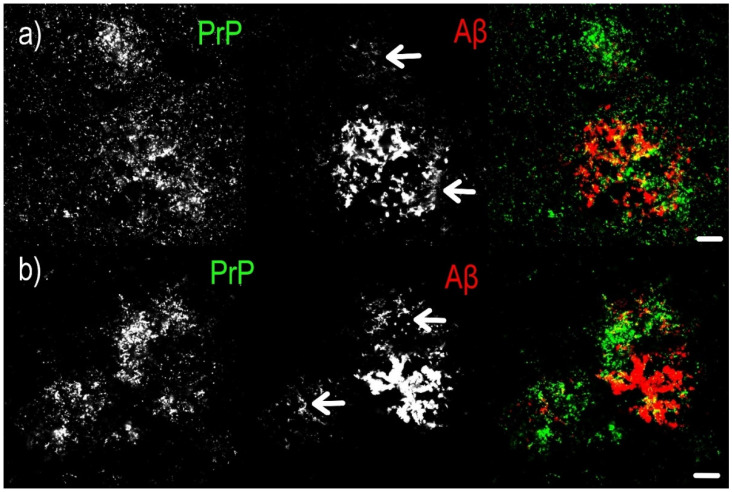
Immunofluorescence visualization and visualization of separated channels showing colocalization of PrP^Sc^ with Aβ in two different (**a**,**b**) non-cored plaques to demonstrate colocalization with more examples. PrP^Sc^ aggregates colocalized predominantly with non-compact Aβ of the senile plaques, while only a minor colocalization was observed in the dense parts. Both images (**a**,**b**) show diffuse “Yin-Yang” compound plaques. Arrows point to non-compact areas of Aβ plaques. Scale bars indicate 10 micrometers.

**Figure 3:**
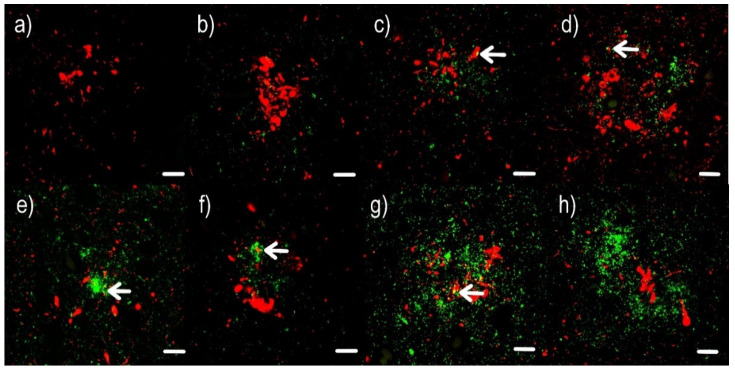
Immunofluorescence visualization of h-tau-positive dystrophic neurites in colocalization with PrP^Sc^ aggregates in comorbid AD and CJD cases (**a**–**h**). Primary antibodies: PrP (rabbit recombinant monoclonal antibody) + AT8 (mouse monoclonal antibody). The secondary antibody was conjugated with either Alexa Fluor^®^ 488 (anti-rabbit IgG, green) or Alexa Fluor^®^ 568 (anti-mouse IgG, red). Scale bars indicate 10 micrometers. Arrows indicate minor colocalization of AT8 with PrP. Images come from the hippocampal region (archicortical parts).

Using confocal fluorescent microscopy, we observed relatively rare compound plaques with either Aβ or hyperphosphorylated tau protein (h-tau) in colocalization with PrP^Sc^. In co-aggregation with Aβ, PrP^Sc^ aggregates colocalized mainly with the non-compact (diffuse) regions of Aβ plaques, while only minor colocalization was observed in the dense regions (see Figure 1 and Figure 2). In contrast, no colocalization between h-tau and PrP^Sc^ was observed, except for a few dot-like colocalizations (see Figure 3).

Visualization of comorbid CJD and AD cases revealed that plaques varied with regard to the micromorphologies of Aβ with PrP^Sc^ between patients. In all subjects, one particular type of Aβ and PrP^Sc^ colocalization predominated. The main types of Aβ and PrP^Sc^ plaque colocalizations identified were (Figure 1):

(1) Non-compound and minimal compound plaques (10 cases out of 17):
  - Non-compound plaques (observed in 3 cases out of 17) are without co-occurrence or colocalization of Aβ and PrPSc deposits. Pure Aβ and pure PrPSc plaque exist independently of each other (Figure 1a,b).
  - Minimal compound plaques (Figure 1c,d) were seen most often (7 of 17 patients in whom PrPSc aggregates were present in the neocortical and archicortical parts of the hippocampal region). The most prominent feature of minimal compound plaques was Aβ (in the form of non-cored or cored plaque); however, dotted PrPSc-immunoreactivities were also present.
(2) Central core deposits—this pattern occurred in both non-cored and cored Aβ plaques (3 cases out of 17). In these cases, a rather significant PrPSc positivity was observed in central non-compact Aβ plaque structures, either with or without dense Aβ cores (Figure 1e–h).
(3) Diffuse plaques (4 cases out of 17):
  - Diffuse compound plaques (Figure 1i,j) contain PrPSc diffusely scattered in the periphery of condensed Aβ plaques and are colocalized with surrounding non-compact Aβ (seen in a single case).
  - Diffuse so-called “Yin Yang” compound plaques (Figure 1k,l; Figure 2) are a particular subset of asymmetric diffuse compound plaques in which PrPSc-positive structures are polarized to the sides of asymmetric plaques in colocalization with non-compact Aβ periphery (i.e., non-compact Aβ in colocalization with PrPSc, but not surrounding the entire circumference of the compact Aβ core aggregates). This type of plaques was observed in 3 cases out of 17.

Similar to colocalization with Aβ in plaques, minimal dot-like colocalization with h-tau positive dystrophic neurites in plaques was recorded (Figure 3).

**Chart 1:**
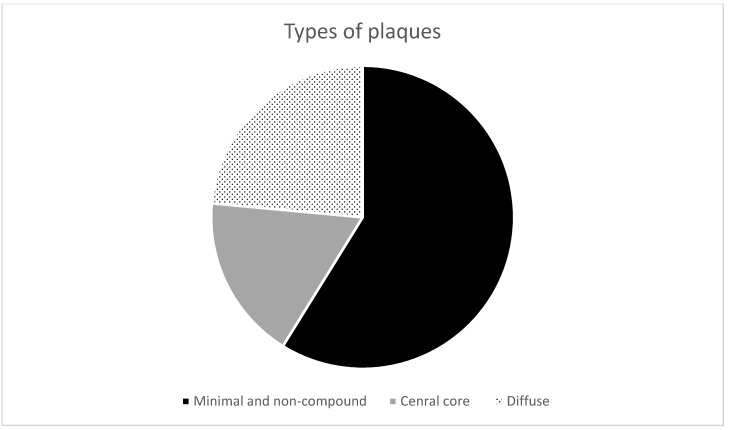
Relative rate of individual types of Aβ and PrP^Sc^ colocalization in plaque.

**Chart 2:**
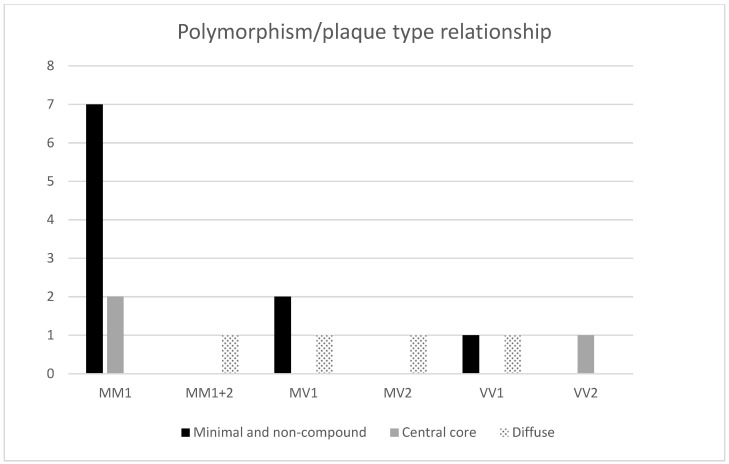
Relationship between methionine–valine (M/V) polymorphisms and plaque type.

**Chart 3:**
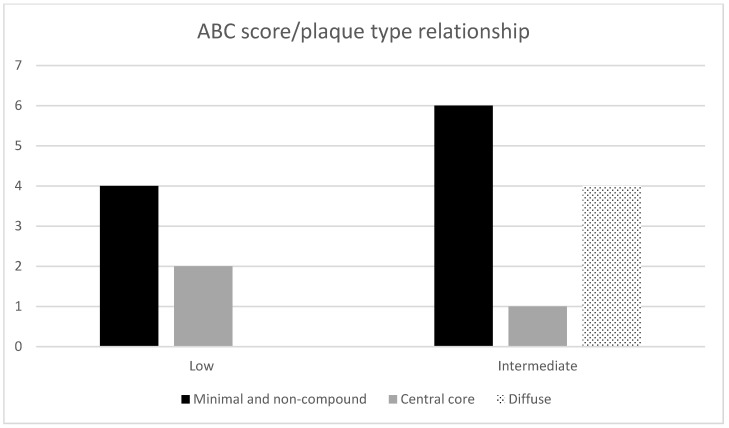
Dependence of plaque-type on the ABC scores.

We analyzed the density and type of colocalization relative to the biochemical and neuropathological properties of CJD and AD; however, no association between plaque micromorphology, type of colocalization, polymorphism at codon 129, type of PrP^Sc^, and the AD ABC score according to the NIA-Alzheimer’s association guidelines was observed (Chart 1, Chart 2 and Chart 3).

### 2.2. Immunohistochemical Examination

**Figure 4:**
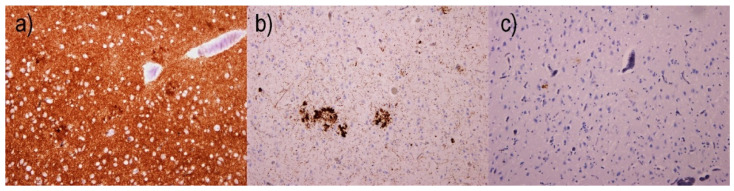
Immunohistochemical imaging of (**a**) PrP^Sc^-positive plaque-like structures, (**b**) AT8-positive dystrophic neurites, and (**c**) Aβ plaques in the same hippocampal region (archicortical part) of an 80-year-old female patient. There are PrP^Sc^ “plaque-like” deposits and h-tau positive neuritic plaques in the same area lacking the equivalent of Aβ plaques. The secondary antibody was conjugated with horseradish peroxidase staining DAB. The original magnification is 100×.

In the immunohistochemical examination, parallel imaging of PrP, hyperphosphorylated tau protein (AT8), and Aβ aggregates in the same hippocampal area (Figure 4a,c) clearly showed the occurrence of PrP^Sc^-positive aggregates in plaques with h-tau positive dystrophic neurites where Aβ was entirely missing. Thus, this observation also suggests that PrP^Sc^ could be colocalized with h-tau-positive dystrophic neurites in Aβ plaques in the absence of Aβ structures. 

## 3. Discussion

In the study presented, a particular affinity of PrP^Sc^ for the non-compact parts of Aβ plaques, suggesting that different subspecies of Aβ have different affinities for PrP^Sc^ aggregates, was observed. In addition, previously reported low rate of compound plaques colocalizing with Aβ or h-tau and PrP^Sc^ in comorbid AD and sCJD were considered.

The potential interaction between PrP^Sc^ and Aβ in comorbid AD and sCJD remains controversial. No significant correlation has been reported for variables influencing the development of CJD and variables determining the course of AD [22]. On the other hand, colocalization of PrP^Sc^ and Aβ in single plaques was reported in a patient with a rare prion disease called Gerstmann–Sträussler–Scheinker disease (GSS) [23]; however, this colocalization was only observed in GSS and not in sCJD [24]. On the contrary, other authors reported compound PrP^Sc^ and Aβ plaques in 11 of 12 evaluated subjects with a concomitant sCJD and AD pathology; the frequency of compound plaques ranged between 2 and 29% [25]. Others also documented the presence of PrP^C^ in senile plaques in non-sCJD AD describing dot-like Pr^C^ -immunoreactivity in diffuse plaques, isolated large coarse PrP^C^-positive structures in neuritic plaques, and dense non-compact or amorphic aggregates in amyloid cores of senile plaques [26].

As mentioned above, the colocalization of Aβ and PrP^Sc^ in different prionopathies in comorbidity with AD was reported, and the frequency of these compound plaques was demonstrated. On the contrary, this study was focused on the detailed micromorphological relationship of PrP^Sc^, Aβ, and AT8 in compound plaques based on confocal fluorescent microscopy.

Based on this, we recognized three types of Aβ and PrP^Sc^ colocalization, as follows: (1) no or minimum compound plaques, (2) compound plaques with a centrally dense PrP^Sc^ core only, and (3) compound plaques with or without a centrally dense PrP^Sc^ core; however, PrP^Sc^ was always present in the periphery. Our data show no or minimal colocalization in the compact region of Aβ plaques; however, in the non-compact region of Aβ plaques, which were often located in the periphery, showed a higher rate of colocalization. Non-compact Aβ plaques are mostly composed of Aβ_42_, rather than Aβ_40_ [27]. Similar to a study on single and double immunohistochemical stains of PrP^C^ in Aβ plaques in AD patients [26], we observed an abundance of dot-like aggregates in diffuse plaques, while in neuritic plaques, PrP deposits tended to be relatively coarse. Interestingly, there seems to be no association between plaque micromorphology, polymorphism at codon 129, type of PrP^Sc^, and the AD ABC scores.

In addition, AT8 positive dystrophic neurites were observed in colocalization with PrP^Sc^ plaque-like structures. Dystrophic; dilated; and, in specific locations, bulbous dystrophic neurites are a common feature of Aβ plaques [21] in AD. Thus, it is not surprising that these structures, which were detected by immunohistochemical positivity of hyperphosphorylated tau protein, are also observed in colocalization with PrP^Sc^. However, using simple DAB immunohistochemistry, we were able to find hippocampal regions with PrP^Sc^ plaque-like positivity and h-tau-positive Aβ plaques without the expression of Aβ (Figure 4), suggesting that direct interaction between PrP^Sc^ and dystrophic neurites or directly with h-tau. Direct molecular interactions between PrP^Sc^ and tau protein have also been reported [28]. Conversely, tau pathology presented as neurofibrillary tangles was a pathognomonic finding for the Indiana Kindred variant of GSS [29]. In the P105L variant of GSS, dystrophic, tau-positive neurites and neurofibrillary tangles were observed in PrP^Sc^ plaques, even in the absence of senile Aβ amyloid plaques [30]. Associations between PrP^Sc^ plaques and tau aggregation were observed in scrapie-infected mouse brains of human tau transgenic mice [31]. Thus, direct pathological interactions between PrP^Sc^ and tau in the absence of Aβ facilitating the progression of the disease could be a plausible hypothesis for AD and sCJD comorbidity. However, our observations are based on a pilot study with a small cohort of patients, and lack a control group with separate AD and CJD cohorts. The results are sustainable for further investigation.

## 4. Materials and Methods

### 4.1. Patients

A total of 20 patients diagnosed with comorbid AD and CJD (age range 62–83 years, median age 71 years) were neuropathologically defined using the National Institute on Aging Alzheimer’s Association (NIA-AA) consensus scheme [32]. Additionally, the presence of PrP^Sc^ in brain tissue was confirmed by both Western-blot and immunohistochemistry. The patient characteristics are summarized in Table 1, which includes gender, age, disease duration, the AD ABC score, [33] codon 129 methionine and/or valine polymorphisms, PrP^Sc^ [34] isoform (i.e., type 1 or 2), specification of other vascular and age-related co-pathologies, and the presence of protein 14-3-3 in the cerebrospinal fluid.

All data were analyzed with respect to patient privacy, and the study was conducted in accordance with the Ethics Committee of Thomayer University Hospital (No G-19-18) on 10 April 2019.

### 4.2. Tissue Samples

Brain tissue samples were fixed for 3–4 weeks in buffered 10% formalin. Then, selected tissue blocks, using a standardized protocol BrainNet Europe [35], were embedded in paraffin using an automatic tissue processor. Five-μm-thick sections were prepared and stained with hematoxylin–eosin, Klüver–Barrera, and silver impregnation methods. For analysis, representative blocks of the left hippocampal and parahippocampal areas were chosen.

### 4.3. Immunofluorescence and Immunohistochemistry

Briefly, 5-μm-thick sections of formalin-fixed and paraffin-embedded tissue samples were deparaffinized and then incubated with primary antibodies for 20 min at room temperature. For Aβ and PrP^Sc^ antibody staining, 96% formic acid was applied prior to the primary antibody. A second layer for light microscopy visualization, consisting of secondary horseradish peroxidase-conjugated antibody (En Vision FLEX/HRP, Dako M822, Glostrup, Denmark), was applied for 20 min at room temperature. The samples were then incubated with DAB (Substrate—Chromogen Solution, Dako K3468, Glostrup, Denmark) for 10 min to visualize the reaction. Mayer’s Hematoxylin Solution was used as a counterstain.

For confocal microscopy, secondary antibodies conjugated to Alexa Fluor^®^ (see below) were used. Paraffin sections were also treated with 20X TrueBlack^®^ (Biotium 23007, Fremont, CA, USA) diluted in 1X 70% alcohol to quench lipofuscin autofluorescence.

#### 4.3.1. Primary Antibodies

For immunohistochemistry, 5-µm-thick sections of formalin-fixed and paraffin-embedded tissue were selected from the hippocampal region, including the entorhinal and transentorhinal cortex. These were incubated with primary antibodies against the following antigens: (1) Aβ (1:1000, mouse monoclonal, clone 6F/3D; Dako M0872, Glostrup, Denmark), (2) Aβ (1:5000, rabbit monoclonal, clone H31L21; Thermo Fisher Scientific 700254, Waltham, ME, USA), (3) PrP (1:8000, mouse monoclonal, clone 12F10; Bertin Pharma A03221, Bordeaux, France), (4) PrP (1:3000, mouse monoclonal, clone 6H8; Prionics 7500996, Schlieren, CH), (5) PrP (1:5000, rabbit recombinant monoclonal, clone SC57-05; Thermo Fisher Scientific MA5-32202, Waltham, ME, USA), and (6) Phospho-Tau (Ser202, Thr205) Monoclonal Antibody (1:500, mouse monoclonal, clone AT8; Thermo Fisher Scientific MN1020, Waltham, ME, USA).

#### 4.3.2. Secondary Antibodies

Detection of immunostaining was carried out using horseradish peroxidase–diaminobenzidine (see above) for immunohistochemistry and secondary antibodies conjugated with Alexa Fluor^®^ 488 (1:1000, donkey anti-rabbit, H + L IgG, Thermo Fischer Scientific, Waltham, MA, USA) and Alexa Fluor^®^ 568 (1:1000, donkey anti-mouse, H + L IgG, Thermo Fischer Scientific) for immunofluorescence staining. Slides incubated with only the secondary antibody were used as specificity controls.

### 4.4. Microscopy Evaluation

#### 4.4.1. Light Microscopy

The samples were examined independently by two neuropathologists focused predominantly on the archicocortical parts of hippocampal region, and the presence/absence of Aβ deposits and AT8-positive structures, in relation to PrP deposits, was evaluated. An Olympus BX51 microscope (Olympus Europa SE and Co. KG, Hamburg, Germany) was used for examination with 100× magnification. Images were captured with an Olympus DP72 camera controlled using Olympus image analysis software (Olympus Europa SE and Co. KG).

#### 4.4.2. Confocal Microscopy

Colocalization of pathogenic protein aggregates was imaged using a Leica TCS SP5 confocal fluorescent laser scanning microscope (Leica Microsystems Inc., Wetzlar, Germany). The HCX PL APO objective with 40× magnification, oil immersion, and a pinhole of 1 AU was used. Donkey anti-Rabbit IgG secondary antibody was conjugated to Alexa Fluor^®^ 488 and excited at 488 nm using a 65 mW multi-line argon laser, whereas Donkey anti-Mouse IgG conjugated to Alexa Fluor^®^ 568 was excited at 561 nm using a 20 mW DPSS laser.

#### 4.4.3. Classification of Aβ Plaques

Diffuse, neuritic non-cored and neuritic cored Aβ plaques were classified according to the literature, as previously summarized in a review article [20].

## 5. Conclusions

The results as presented indicate that a specific subset of Aβ, in particular the non-compact component of Aβ plaque where Aβ_42_ predominates, exhibits higher levels of interaction with PrP^Sc^ and, thus, in certain circumstances, could be assumed to act as the PrPSc seeds within the brain (see Supplementary Materials). The role of PrP^Sc^ in the development of neuritic plaques, with or without the Aβ component, certainly requires further investigation.

## Figures and Tables

**Table 1:** Individual patients, including gender, age, duration of the disease, 129 codon polymorphisms with the PrP type, 14-3-3 positivity, the AD ABC score according to the NIA-Alzheimer’s association guidelines, data regarding other neuropathologies, and plaque type specification.

No.	Sex	Age	Duration (Months)	Polymorph.	PrP Types Brain Tissue	Western Blot (14-3-3) CSF	AD ABC Score	Other Neuropathology	Type of Plaques
1.	M	75	2	MM	1	neg.	A2B2C2	Angiosclerotic encephalopathy, ARTAG, encephalomalacia	Non-compound
2.	F	71	1	MV	1	low pos.	A2B1C2	Angiosclerotic encephalopathy	Non-compound
3.	F	75	9	VV	1	pos.	A1B1C1	Angiosclerotic encephalopathy	Non-compound
4.	F	67	1	MM	1	pos.	A2B1C1	Angiosclerotic encephalopathy	Minimal compound
5.	F	69	2	MM	1	NA	A2B2C1	Angiosclerotic encephalopathy	Minimal compound
6.	M	62	6	MV	1	neg.	A3B2C2	Angiosclerotic encephalopathy	Minimal compound
7.	F	65	2	MM	1	pos.	A2B2C2	Angiosclerotic encephalopathy	Minimal compound
8.	M	79	1	MM	1	pos.	A1B2C1	Angiosclerotic encephalopathy	Minimal compound
9.	M	75	4	MM	1	neg.	A2B2C2	Angiosclerotic encephalopathy, ARTAG, Wernicke encephalopathy, meningioma	Minimal compound
10.	F	68	2	MM	1	pos.	A2B2C2	Angiosclerotic encephalopathy, AGD	Minimal compound + few compounds
11.	M	79	1,5	MM	1	pos.	A2B2C2	Angiosclerotic encephalopathy, ARTAG	Central core deposits
12.	F	71	1	MM	1	pos.	A2B1C2	Angiosclerotic encephalopathy	Central core deposits
13.	F	80	2	VV	2	pos.	A3B1C2	Angiosclerotic encephalopathy	Central core deposits
14.	M	64	2	MV	2	low pos.	A2B2C2	Angiosclerotic encephalopathy, Wernicke encephalopathy	Diffuse compound
15.	F	70	2	VV	1	pos.	A2B2C2	Angiosclerotic encephalopathy	Yin-Yang
16.	F	70	1	MM	1 + 2	pos.	A2B2C3	Angiosclerotic encephalopathy	Yin-Yang
17.	F	80	5	MV	1	pos.	A3B2C3	Angiosclerotic encephalopathy	Yin-Yang + few compounds
18.	M	83	1	MM	1	low pos.	A2B2C2	Angiosclerotic encephalopathy, ARTAG	Lacking PrP plaques in the hippocampus
19.	F	65	5	VV	1	pos.	A1B1C1	Angiosclerotic encephalopathy	Lacking PrP plaques in the hippocampus
20.	M	85	2	MM	1	neg.	A2B2C2	Angiosclerotic encephalopathy	Lacking PrP plaques in the hippocampus

M—male; F—female; MM/MV/VV—methionine and/or valine polymorphism on codon 129; CSF—cerebrospinal fluid; ARTAG—aging-related tau astrogliopathy; AGD—argyrophilic grain disease; PrP—prion protein.

## Data Availability

The authors confirm that all data underlying the findings are fully available without restriction. All data are included within the manuscript https://doi.org/10.3390/ijms22042099.

## Supplementary Materials

The following are available online at https://www.mdpi.com/1422-0067/22/4/2099/s1.
