# Supplementary material for: Extracellular Protein Aggregates Colocalization and Neuronal Dystrophy in Comorbid Alzheimer’s and Creutzfeldt–Jakob Disease: A Micromorphological Pilot Study on 20 Brains
Source: Int J Mol Sci. 2021 Feb 20;22(4):2099. doi: 10.3390/ijms22042099 (PMC7924045; doi:10.3390/ijms22042099)

Supplementary Materials

Scheme of presumed seeding of PrP^{Sc} in non-compact parts of Aβ plaques in AD and CJD in comorbidity. It is hypothesized that the first step in the development of the compound Aβ and PrP^{Sc} plaques is formation of either cored or non-cored neuritic plaques where the compact parts are enriched by $A\beta_{40}$, while non-compact parts with predominant $A\beta_{42}$, is located mostly in the periphery. The second step is coaggregation of pathological PrP^{Sc} not only, but mostly in the non-compact periphery of either cored or non-cored neuritic plaques. The scheme was composed using Servier Powerpoint Image Bank: http://www.servier.com/.

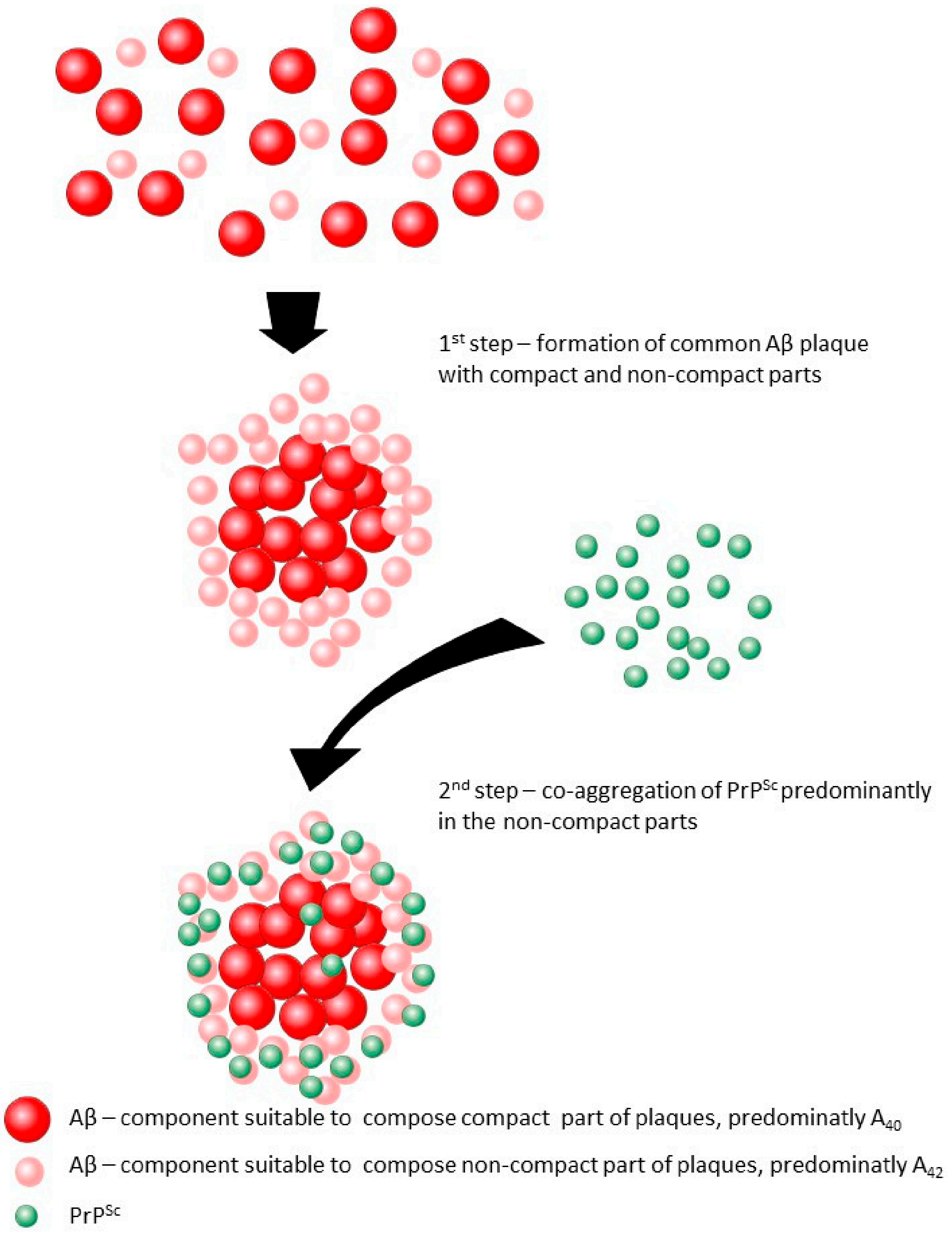

Supplement: Supplementary file 1 [file ijms-22-02099-s001.pdf]
